# Hand Grip Strength, Osteoporosis, and Quality of Life in Middle-Aged and Older Adults

**DOI:** 10.3390/medicina59122148

**Published:** 2023-12-11

**Authors:** Hyo Jin Park, Byoungduck Han, So-youn Chang, Seung Ho Kang, Dae Wook Lee, Seok Kang

**Affiliations:** 1 Department of Family Medicine, Korea University Guro Hospital, Korea University College of Medicine, Seoul 08308, Republic of Korea; geeni14022@gmail.com; 2 Department of Family Medicine, Korea University Anam Hospital, Korea University College of Medicine, Seoul 02841, Republic of Korea; arybury1@naver.com; 3 Department of Rehabilitation Medicine, Yeouido St. Mary’s Hospital, College of Medicine, The Catholic University of Korea, Seoul 07345, Republic of Korea; jsy870930@gmail.com; 4 Department of Medical Device Industry, Dongguk University, Seoul 04620, Republic of Korea; rnd3@dsmaref.com; 5 Department of Rehabilitation Medicine, Korea University Guro Hospital, Seoul 08308, Republic of Korea; daewoogi95@korea.ac.kr

**Keywords:** hand grip strength, osteoporosis, quality of life, muscle strength, mental health, EQ-5D

## Abstract

*Background and Objectives*: Hand grip strength (HGS) and osteoporosis are known to be closely related to the health condition of the elderly, respectively. Comprehensive studies including adults over middle age were insufficient. This study aimed to investigate the relationship between HGS with osteoporosis and health-related quality of life (HRQoL) in adults aged >40 years. *Materials and Methods*: This cross-sectional analysis included data from 13,966 people aged >40 years between 2015 to 2018 provided by the Korea National Health and Nutrition Examination Survey. The HGS was divided into strong and weak quartiles, defined as the highest and lowest quartiles, respectively. We used the European Quality of Life Scale-Five dimensions (EQ-5D) for HRQoL. We performed multiple logistic regression and post hoc analysis to confirm the relationship between the four groups and HRQoL. *Results:* Osteoporotic patients with weak HGS showed the lowest EQ-5D index (0.87 ± 0.01) among all groups and had a significantly impaired HRQoL in all EQ-5D dimensions, at least 1.75 times more than healthy individuals with strong HGS (0.95 ± 0.00). Osteoporotic patients with weak HGS showed, notably, 2.68 times more impaired mobility compared to healthy individuals with strong HGS among all five dimensions of the EQ-5D. In self-care, significant sex differences in impaired HRQoL were observed (males 6.03, 2.23–16.35; females 2.51, 1.70–3.71). *Conclusions*: Weak HGS and the presence of osteoporosis were associated with low HRQoL, respectively. Middle-aged and older adults with both weak HGS and osteoporosis showed poorer HRQoL compared to healthy middle-aged and older adults. This suggests that HGS is a possible factor for predicting poor HRQoL in adults aged >40 years with or without osteoporosis. It is necessary to assess the risk of low HRQoL by measuring HGS and confirming whether osteoporosis is accompanied in adults over middle age.

## 1. Introduction

Handgrip strength (HGS) has garnered considerable attention as a biomarker for various health statuses in adults, including the elderly [1]. Primarily, HGS serves as an indicator of muscle mass or strength, and a weak HGS has been associated with sarcopenia [2]. Sarcopenia appears during the aging process associated with the musculoskeletal system and is an important problem accompanied by significant loss of physical performance [3]. It is known that with external stimulation, such as motivation, physical abilities can be improved more than without stimulation [4]. However, physical activity above a certain level may accelerate this aging process more rapidly [3]. Many studies have shown that weaker HGS is associated with low bone mass density (BMD), a higher risk of osteoporosis and fractures, and reduced bone health, which is important for adult health [5]. Additionally, HGS is not only correlated with early all-cause or cardiovascular disease-related mortality but also serves as a valuable tool for assessing nutritional status and fall risk [6,7]. The simplicity of measuring HGS using a hand grip dynamometer contributes to its widespread use as an indicator for various health conditions [8]. In South Korea, HGS has been measured in people aged 10 years and older since 2014 in the National Health and Nutrition Examination Survey (KNHANES) due to its ease of measurement and representativeness as a health indicator [9,10].

The elderly experience mental health problems, such as anxiety about death and low-quality sleep, while also facing physical health issues like osteoporosis [11]. Osteoporosis is a critical public health concern, with an increased mortality risk following osteoporotic fractures in the elderly population [12]. Numerous studies have evaluated the association between health-related quality of life (HRQoL) and osteoporosis, which is generally found to be negatively impacted [13]. Diagnosis of osteoporosis typically involves the use of dual-energy X-ray absorptiometry (DEXA) to measure the T-score of an individual’s bone mineral density (BMD) in the whole femur, femoral neck, and lumbar spine [14]. BMD measured by DEXA is related to HGS, and the grip strength of the dominant hand can be a BMD predictor [15]. However, compared with the relatively straightforward measurement of HGS in clinical practice, DEXA is more complex [8,16]. Therefore, given that HGS is known as a great tool for assessing diverse health statuses [6], research on the association between HGS and HRQoL in individuals with or without osteoporosis holds practical importance [17,18,19].

In middle-aged adults, fractures and osteoporosis are less prevalent, but occur in the presence of chronic diseases, drugs that influence bone metabolism, and other risk factors [20]. Osteoporosis is difficult to diagnose and treat in adults aged <50 years [21]. In a previous study, HGS was found to be a key factor influencing radial BMD in the dominant forearm of young people [22]. Other previous studies explored the relationship between HGS and HRQoL, or osteoporosis and HRQoL in older adults [6,13]. However, few studies have compared and analyzed this association in middle-aged individuals aged over 40 years, including older adults, with or without osteoporosis in Asian populations. 

Therefore, we hypothesized that individuals with weaker HGS and the presence of osteoporosis would exhibit a more pronounced decline in HRQoL compared to those with stronger HGS and without osteoporosis. We aimed to explore the relationship between HGS with or without osteoporosis and HRQoL in middle-aged and older adults aged >40 years using a nationwide cross-sectional database from the Republic of Korea. We divided the participants into four groups based on relative levels of HGS and the presence or absence of osteoporosis. In addition, to confirm sex differences, we applied the same classification of participants to both men and women and conducted the analysis.

## 2. Materials and Methods

### 2.1. Data Source

This study used raw data collected between 2015 and 2018 provided by KNHANES in the Republic of Korea. This survey has been conducted nationally and yearly since 1998 by the Korea Centers for Disease Control and Prevention (KCDC). The KCDC directly selects survey participants among Koreans to ensure they represent the characteristics of the entire Korean population. Each year, this survey is conducted with approximately 10,000 new participants by KCDC [23]. The KNHANES is a national survey, and participants voluntarily took part in the study with informed consent. Our research used the data from KNHANES, which are publicly available. Data were collected through a self-report questionnaire, an individual survey for health information, a medical examination, and a nutritional survey to examine people’s health status, including health behaviors, nutritional intake conditions, and mental health status [13]. All processes of this survey were conducted by professional investigators following accurate protocols predetermined in advance. These investigators underwent high-intensity training courses beforehand and carried out actual investigations under the supervision of supervisors [23]. In 2016 and 2017, the study was conducted without deliberation according to the opinions of the Institutional Review Board of the KCDC. In 2015 and 2018, data were collected from studies conducted with the approval of the Institutional Review Board of the KCDC (No. 2015-01-02-6C, No. 2018-01-P-A) [24].

### 2.2. Study Population

**Figure 1:**
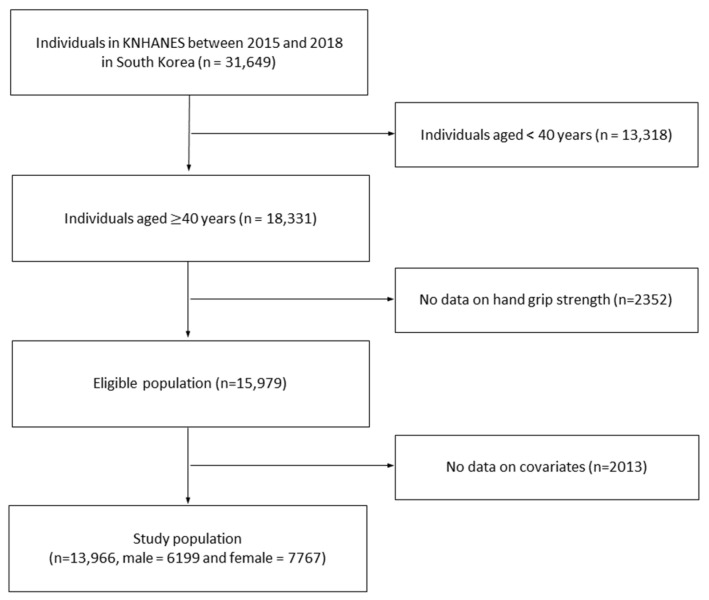
Flow diagram of the procedure to select the study population in our study. Abbreviations: KNHANES, the Korea National Health and Nutrition Examination Survey.

As shown in Figure 1, between 2015 and 2018, 31,649 people participated in the survey. We included only those over 40 years of age and excluded those with missing values, including HGS, leaving 13,966 participants. The presence of osteoporosis was defined based on the answer of “Yes” to the question “Have you been diagnosed with osteoporosis by a physician?” among standardized self-report questionnaires in the KNHANES. In our study, we divided the final population into two groups: those with and without osteoporosis. Those without osteoporosis were referred to as healthy individuals and those with osteoporosis were referred to as osteoporotic patients.

### 2.3. Measurement of HGS

HGS was measured using a digital grip strength dynamometer (T.K.K 5401; Takei Scientific Instruments Co., Ltd., Tokyo, Japan). During the assessment, the participants were asked to stand upright with their feet hip-width apart and look forward with the elbow fully extended. The dynamometer was held by the testing hand in a neutral, comfortable position (not flexed or extended) with 90° flexion at the index finger. Participants alternately performed three trials for each hand, starting with the dominant hand. The participants were instructed to squeeze the grip continuously with full force for at least 3 s and were asked not to swing the grip dynamometer during the test and not to hold their breath [2]. A rest interval of at least 30 s was allowed between the measurements [25]. HGS was defined as the maximum grip strength among all three measurements. The participants were divided into two groups according to the level of HGS: strong and weak HGS, which were defined as the highest and lowest quartile, respectively. The lowest HGS had a cutoff value of 33.3 and 19.8 kg for men and women, respectively [26].

The measurement of HGS was conducted before the participants underwent a blood test, as they needed to exert their maximum instantaneous force. The aforementioned HGS measurement process was carried out by experienced professional investigators who underwent training in HGS measurement to enhance the accuracy of the measurements. These investigators received detailed training in the process of HGS measurement through biannual sessions in KCDC. The digital dynamometer was inspected daily before use to minimize errors in the measurement process.

### 2.4. Assessment of HRQoL

To assess HRQoL, we used the European Quality of Life Scale-Five dimensions (EQ-5D) developed by the EuroQol Group. The EQ-5D self-report questionnaire comprises five dimensions of health: mobility, self-care, usual activities, pain/discomfort, and anxiety/depression, with three possible answers to each question (1 = no problem, 2 = moderate problem, 3 = severe problem) [27]. The EQ-5D index scores, which represent HRQoL, generally range from less than 0 (equivalent to health; negative values indicate a worse status than death) to 1 (perfect health). Therefore, the higher the EQ-5D index score, the higher the HRQoL [28]. We used the EQ-5D index with the quality weight estimated for Koreans, as reported by the KCDC [29].

### 2.5. Definition of Covariates

Information on participants’ demographics and health-related behaviors was collected using a standardized self-report questionnaire from the KNHANES. Participants considered to be at the low-income level were defined as those receiving medical aid from the government or who had a household income in the bottom 20% of the population. Education level was based on the total education period of 10 years. Smoking status was divided into three categories: non-, ex-, and current smokers. Non-smokers were defined as those who smoked fewer than five cigarettes in the past, and ex-smokers were defined as those who smoked more than five cigarettes in the past but were not currently smoking. According to the World Health Organization (WHO) standards, heavy drinking is defined as drinking more than seven drinks at a time for men or more than five drinks for women twice a week. Drinking less than this standard was defined as mild. Aerobic physical activity was defined as performing moderate-intensity exercise for ≥5 d/week or vigorous-intensity exercise for ≥3 d/week. In addition, the presence or absence of hypertension, diabetes mellitus, and hypercholesterolemia were identified using a questionnaire regarding whether individuals currently had these comorbid diseases. Height, weight, waist circumference (WC), and blood pressure were measured, and the body mass index (BMI) was calculated by dividing the participants’ weight (kg) by the square of their height (m). Laboratory test results included fasting serum glucose, total cholesterol, and high-density lipoprotein cholesterol (HDL-C).

### 2.6. Statistical Analysis

Statistical analyses were performed using SAS software (version 9.4; SAS Institute, Cary, NC, USA). To compare demographic and basic characteristics, continuous variables were compared as average values using the independent *t*-test, and in the case of categorical variables, percentages (%) were expressed using the chi-square test. The subgroups were divided and analyzed according to the diagnosis of osteoporosis and HGS. Model 1 was not adjusted. In Model 2, variables for sex, age, economic level, education level, smoking status, drinking status, and physical activity were adjusted. Model 3 was further adjusted for diabetes, high blood pressure, and hypercholesterolemia in Model 2. Analysis of covariance (ANCOVA) was used to compare the average EQ-5D index scores for each subgroup. The Bonferroni correction was used for the post hoc analysis of this result, and the significance level was adjusted to *p* < 0.0083. Multiple logistic regression analysis was used to calculate odds ratios (OR) for healthy individuals with strong HGS versus other groups to identify the association between weak HGS and low health-related HRQoL. Statistical significance was set at *p* < 0.05, and 95% confidence intervals (CI) were presented for ORs, which are shown in forest plots.

## 3. Results

### 3.1. Baseline Characteristics of the Total Participants

Table 1 shows a comparison of the basic demographic and health characteristics between the two groups according to the intensity of HGS. Of the final 13,966 participants, 6199 and 7767 were men and women, respectively. The number of participants in the strong HGS group was 10,450, which was more than that in the weak HGS group (*n* = 3516). The average HGS in the strong HGS group was 34 and that of the weak HGS group was 21. The osteoporosis diagnosis rate was 6.27% in the strong HGS group and 16.15% in the weak HGS group. The average age of the weak HGS group was 64 years, which was higher than that of the strong HGS group (54 years). The proportion of participants with more than 10 years of training was higher (72%) in the strong HGS group. The proportion of proper physical activity was 45.52% in the strong HGS group, which was higher than that in the weak HGS group (33.03%). The proportion of people with comorbid diseases was generally higher among those with weak HGS. The average EQ-5D index score was 0.96 in the strong HGS group and 0.9 in the weak HGS group. In each of the five dimensions of the EQ-5D, the rate of having a problem or feeling discomfort was higher in the weak HGS group than in the strong HGS group.

### 3.2. HGS in Participants with or without Osteoporosis

Table 2 shows HGS according to the presence of osteoporosis by sex in the three models. Of the total participants, 12,465 were healthy individuals without osteoporosis and 1501 were patients with osteoporosis. In all participants, the average values of HGS between healthy individuals who do not have osteoporosis and the group of osteoporotic patients in Model 3 differed significantly, but the difference was quite small.

### 3.3. The Comparison of EQ-5D Index by Subgroup Analysis

Table 3 shows the results of the comparison of EQ-5D values according to HGS and the presence of osteoporosis. In all models, all osteoporotic patients with weak HGS showed the lowest EQ-5D values among the four groups. Similarly, healthy individuals with strong HGS showed the highest EQ-5D values among the four groups. A Bonferroni post hoc analysis was conducted to compare the EQ-5D between these four groups in Model 3, and the results are shown in Supplementary Table S1. Post hoc analysis revealed that osteoporotic patients with weak HGS showed significantly lower EQ-5D scores than the other three groups. In other words, patients with osteoporosis with weak HGS showed significantly lower HRQoL than those with strong HGS and without osteoporosis.

### 3.4. The Comparison of HRQoL by the Presence of Osteoporosis and HGS

**Figure 2:**
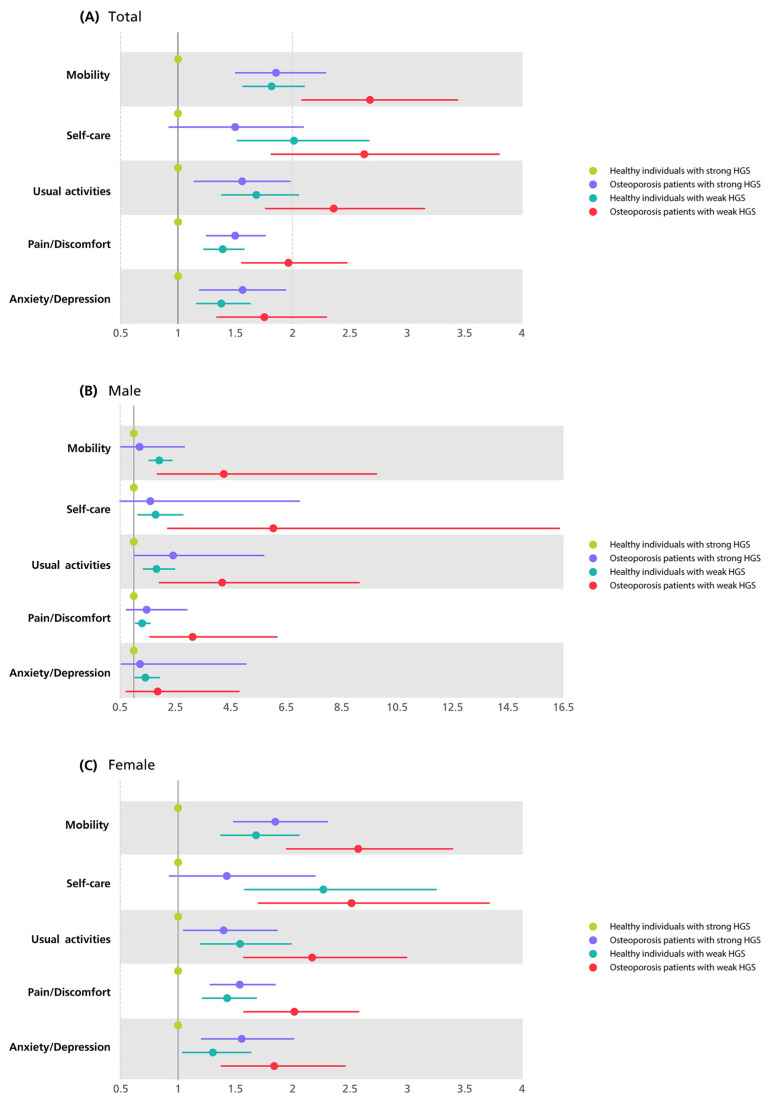
Forest plots of odds ratios of impaired HRQoL by the presence of osteoporosis and HGS in all five dimensions of EQ-5D in total participants (**A**), male (**B**), and female (**C**) in model 3^a^. ^a^ Model 3 was adjusted for gender, age, income, education level, smoking status, drinking status, physical activity, diabetes, hypertension, and hypercholesterolemia. Abbreviations: HRQoL, health-related quality of life; HGS, hand grip strength; EQ-5D, European Quality of Life Scale-Five.

Figure 2 illustrates the ORs for impaired HRQoL in all five dimensions in the other three groups based on the values of healthy individuals with strong HGS in Model 3 using forest plots. Supplementary Table S2 shows the exact ORs of all five dimensions of the EQ-5D in the presence of osteoporosis and HGS. Among the participants in Model 3, osteoporotic patients with weak HGS were 2.68 times more likely to have moderate or severe problems with mobility, 2.63 times higher for self-care, 2.36 times higher for usual activities, 1.96 times higher for pain/discomfort, and 1.76 times higher for anxiety/depression than healthy individuals with strong HGS. In Model 3, compared with healthy individuals with strong HGS, the higher relative risk of poor HRQoL in osteoporotic patients with weak HGS generally showed higher ORs in all dimensions in men than in women.

## 4. Discussion

Our study revealed that individuals with osteoporosis and weak HGS were at least 1.3 times more likely to experience moderate or severe problems in all five HRQoL dimensions than those without osteoporosis and with strong HGS. In other words, people with osteoporosis and weak HGS had significantly poorer HRQoL, regardless of sex, when compared to osteoporotic patients with strong HGS and even healthy individuals with weak or strong HGS.

The average HGS values of both healthy individuals and patients with osteoporosis calculated in our study were generally similar to those found in previous studies on HGS values for the general population in Korea [30]. This similarity may be attributed to the fact that we utilized data from the KNHANES, which are representative of the general Korean population.

In our study, we observed that women with osteoporosis had significantly weaker HGS than those without osteoporosis. This finding is consistent with that of previous research showing a link between HGS and the risk of low BMD and osteoporosis. Studies have indicated that menopausal women with low HGS tend to have lower BMD in areas such as the lumbar spine, femoral neck, and total hip [31].

However, the difference in the HGS between healthy individuals and patients with osteoporosis in our study was not substantial. There are several possible explanations for this observation. Above all, our study included women over the age of 40 years, and there was a higher number of women with strong HGS. This may have influenced the results. Additionally, the number of healthy women in our study was much higher than the number of women with osteoporosis, which may have affected the observed difference in HGS between the two groups.

Through subgroup analysis, we observed a significant difference in the EQ-5D index values among the four groups categorized according to HGS and osteoporosis. Notably, patients with osteoporosis and weak HGS displayed the lowest EQ-5D index (0.87 ± 0.01) compared to the other groups. While this finding does not establish a clear causal relationship, it does indicate that osteoporotic patients with weak HGS tend to have lower HRQoL compared to those with strong HGS (0.95 ± 0.00, 0.93 ± 0.01) or all healthy individuals (0.95 ± 0.00, 0.93 ± 0.00). Given that our study established a definite association between HGS and HRQoL, further research is warranted to investigate the causal relationship between these factors.

In our study, we found that patients with osteoporosis and weak HGS exhibited the highest risk of experiencing moderate or severe problems in all five dimensions of the EQ-5D compared to the other groups. Notably, when analyzing both sexes separately or combined, the risk of deficiencies in the dimensions of mobility and self-care were consistently the highest (2.68, 95%CI 2.08–3.44 and 2.63, 95%CI 1.81–3.80, respectively), followed by the risk (2.36, 95%CI 1.77–3.15) of problems in usual activities. The heightened risks in these three dimensions can be attributed, in part, to the relationship between HGS and muscles. Previous studies have consistently demonstrated a strong association between HGS and muscle mass, showing that weak HGS is often accompanied by reduced muscle mass and diminished physical abilities [32]. Consequently, a weak HGS can be viewed as a predictor of reduced muscle mass and impaired physical function. Additionally, studies have highlighted HGS as an indicator of nutritional status [7], and poor nutritional status has been identified as a risk factor not only for sarcopenia in adults but also for hindering daily activities, such as self-care and usual activities [33,34]. Furthermore, osteoporosis is associated with walking speed, which is related to muscle strength [35]. Thus, in patients with osteoporosis and weak HGS, it can be assumed that slow walking speed and weak muscle strength significantly interfere with the HRQoL related to physical functions, including daily living and mobility [13,36]. Consequently, HGS serves not only as an indicator of muscle strength and bone health but also of impaired HRQoL related to physical function. In the case of people with osteoporosis, physicians should measure their HGS to evaluate whether they have any problems performing their physical functions and basic daily living so that additional problems caused by impaired HRQoL can be prevented in advance.

Considering sex differences in HRQoL, the relative risk of having problems in the three dimensions related to physical function was generally higher in men with osteoporosis and weak HGS than in women. In particular, in the dimension of self-care, the OR of osteoporotic patients with weak HGS in men was 6.03 (95%CI, 2.23–16.35), which was significantly different from the risk of the women 2.51 (95%CI, 1.70–3.71) after adjusting for covariates. There is a difference between men and women in terms of HRQoL, and men are generally known to perform better physical functions than women [37]. Men have weaker HGS and osteoporosis, which are associated with lower HRQoL, especially related to their physical functioning. Thus, monitoring HGS equivalent to that of women may be needed, even in men who are already known to have better physical function.

In the pain/discomfort dimension, we also found that patients with osteoporosis and weak HGS, including both males and females, had a higher risk (overall: 1.96, 95%CI, 1.56–2.47; male: 3.12, 95%CI, 1.58–6.16; female: 2.02, 95%CI, 1.58–2.58) of having moderate or severe problems than the other two groups, even after adjusting for covariates. In the case of pain/discomfort, previous studies have shown that pain reduces muscle strength and the range of muscle exercise [38]. This may affect HRQoL dimensions related to physical function, such as mobility, self-care, and usual activities. In addition, another previous study reported that low muscle strength can lower the threshold of pain; therefore, osteoporotic patients with weak HGS can be more sensitive to pain and their quality of life may be affected in this regard [39].

Previous studies have shown a correlation between low muscle strength and depressive symptoms, particularly in elderly populations [40]. Another study revealed a link between low grip strength and increased stress [41]. Previous studies of not only the elderly but also adults aged 45 years or older, who were similar in age to our study, showed a link between low grip strength and a higher risk of mental illness [42]. These results are consistent with our findings that osteoporotic patients with weak HGS have a significantly higher risk (1.76, 95%CI, 1.34–2.29) of having problems in the anxiety and depression dimensions of HRQoL compared to other groups, even after adjusting for covariates. Previous studies and the results of the present study suggest a relationship between HGS and HRQoL, especially with respect to mental health. First, according to the results of our study, osteoporotic patients with low HGS showed poor physical function and difficulties in their daily living. As shown in previous studies, decreased physical functioning is associated with mental illness [42]. Therefore, it may be inferred that weak HGS, which can reflect decreased bone health or muscle weakness, may be associated with mental symptoms, such as anxiety and depression, induced by deteriorated physical function. However, our findings showed the lowest relative risk (1.75, 95%CI, 1.34–2.29) of having moderate or severe problems in the anxiety/depression dimension, compared to the ORs of the other four dimensions (OR: mobility 2.68, 95%CI, 2.08–3.44; self-care 2.63, 95%CI, 1.81–3.80; usual activities 2.36, 95%CI, 1.77–3.15; pain/discomfort 1.96, 95%CI, 1.56–2.47). This suggests that mental health has a less independent association with impaired HRQoL than physical functioning. According to previous studies, weak HGS is related to mental conditions, such as cognitive decline or depressive symptoms [43,44]. The association between grip strength and mental health can be inferred about the mechanism from the perspective of muscles. In our study and previous studies, it was confirmed that low muscle strength is related to physical inactivity. These physical activities may cause changes in several factors and interleukins secreted by muscles during exercise and may affect other organs, including the brain [45]. Therefore, this may reflect a mental health status in which weak HGS associated with physical inactivity can be reduced.

Our study has some limitations. The diagnosis of osteoporosis may be determined in the form of self-reporting, asking participants whether they have been diagnosed by a physician; there may be limitations in the survey on the accuracy of osteoporosis diagnosis. The main limitation of this study is that it was difficult to prove a causal relationship between HGS, osteoporosis, and HRQoL using a cross-sectional analysis of the relationship between these three factors. Therefore, in the future, observational studies will be needed to determine how weak HGS and the presence of osteoporosis affect changes in HRQoL over time among adults beyond middle age. In addition, Models 2 and 3 considered various variables, such as demographics and comorbid diseases, but it is a limitation that we did not consider other comorbidities that may affect physical function or already existing mental disorders, such as depression, which can affect mental health.

Despite these limitations, this study has several strengths. Many studies have examined the relationship between HGS and muscle strength, bone health, and quality of life, mainly in elderly or menopausal women. However, our study selected a relatively large number of adults over the age of 40 years, including the elderly, who represent the general population. HGS, which has been mainly known as a marker of multiple health status in the elderly, has been confirmed to be an important health indicator even in middle-aged people. Another advantage of our study is that we not only analyzed the relationship between HGS and HRQoL but also considered participants who were diagnosed with osteoporosis at the same time. Among patients with osteoporosis, HGS measurements may be a tool to determine which group is more sensitive to impaired HRQoL. It can also be advantageous to evaluate HRQoL using the EQ-5D, which has been standardized and proven to represent quality of life in many studies [26]. Finally, a strength of our study is that the participants were divided into four groups and compared through subgroup and post hoc analyses.

## 5. Conclusions

In our study, we found a close relationship between weak HGS and poor HRQoL in patients with osteoporosis aged over 40 years. Significant correlations were observed across all physical and mental dimensions of HRQoL. Our research demonstrates that HGS serves as an easily measured and reliable marker of HRQoL in clinical practice. Therefore, physicians should assess HGS in patients with osteoporosis to proactively identify areas that require attention to improve their physical and mental well-being and overall quality of life. Further studies are necessary to establish the causal relationship between HGS with osteoporosis and quality of life.

## Figures and Tables

**Table 1:** Demographic and clinical characteristics of participants with strong and weak HGS (*n* = 13,966).

	Total			Men			Women		
	Strong HGS	Weak HGS	*p*-Value	Strong HGS	Weak HGS	*p*-Value	Strong HGS	Weak HGS	*p*-Value
*N*	10,450	3516		4652	1547		5798	1969	
Osteoporotic patients	6.27 (0.24)	16.15 (0.76)	<0.001	0.73(0.14)	2.54 (0.47)	<0.001	11.91 (0.46)	27.13 (1.26)	<0.001
HGS (kg)	34 ± 0.1	21.82 ± 0.15	<0.001	42.32 ± 0.11	28.46 ± 0.12	<0.001	25.48 ± 0.06	16.39 ± 0.07	<0.001
Age	54.1 ± 0.2	64.4 ± 0.29	<0.001	53.68 ± 0.17	64.73 ± 0.37	<0.001	54.54 ± 0.17	64.13 ± 0.37	<0.001
Low income	316	34.34 (1.21)	<0.001	10.73 (0.56)	31.46 (1.45)	<0.001	15.47 (0.63)	36.69 (1.53)	<0.001
Education ≥10 years	72.39 (0.71)	44.15 (1.24)	<0.001	78.26 (0.79)	52.29 (1.63)	<0.001	66.39 (0.84)	37.48 (1.46)	<0.001
Smoking			<0.001			<0.001			0.304
Non-smoker	56.01 (0.53)	60.59 (0.97)		20.5 (0.7)	20.33 (1.18)		92.3 (0.43)	93.54 (0.69)	
Ex-smoker	24.07 (0.48)	24.18(0.77)		44.36 (0.87)	50.43 (1.44)		3.34 (0.27)	2.68 (0.4)	
Current-smoker	19.92 (0.51)	15.24 (0.82)		35.14 (0.88)	29.24 (1.49)		4.36 (0.35)	3.78 (0.56)	
Drinking			<0.001			<0.001			<0.001
Non	23.85 (0.53)	41.82 (1)		14.92 (0.62)	28.28 (1.42)		32.97 (0.77)	52.9 (1.37)	
Mild	66.41 (0.58)	51.89 (1.05)		67.61 (0.82)	59.16 (1.51)		65.18 (0.78)	45.94 (1.36)	
Heavy	9.74 (0.34)	6.29 (0.51)		17.47 (0.63)	12.56 (1.09)		1.84 (0.23)	1.16 (0.27)	
Aerobic physical activity	45.5 (0.7)	33.03 (1.01)	<0.001	47.21 (0.9)	37.6 (1.61)	<0.001	43.79 (0.8)	29.3 (1.17)	<0.001
Diabetes mellitus	12.9 (0.4)	21.43 (0.83)	<0.001	15.19 (0.59)	23.6 (1.27)	<0.001	10.47 (0.48)	19.66 (1.05)	<0.001
Hypertension	34.7 (0.6)	48.42 (1.15)	<0.001	39.64 (0.83)	47.66 (1.55)	<0.001	29.71 (0.72)	49.04 (1.49)	<0.001
Hypercholesterolemia	25.16 (0.51)	28.68 (0.9)	<0.001	22.84 (0.68)	21.48 (1.22)	0.317	27.54 (0.66)	34.58 (1.22)	<0.001
BMI (kg/m^2^)	24.32 ± 0.04	23.68 ± 0.07	<0.001	24.74 ± 0.05	23.42 ± 0.09	<0.001	23.89 ± 0.06	23.88 ± 0.09	0.990
WC (cm)	83.75 ± 0.12	83.22 ± 0.2	0.014	87.23 ± 0.14	85.33 ± 0.27	<0.001	80.2 ± 0.16	81.49 ± 0.26	<0.001
SBP (mmHg)	120.13 ± 0.22	123.82 ± 0.41	<0.001	121.88 ± 0.28	123.18 ± 0.52	0.019	118.34 ± 0.29	124.34 ± 0.56	<0.001
DBP (mmHg)	77.85 ± 0.13	73.96 ± 0.23	<0.001	80.25 ± 0.18	74.24 ± 0.33	<0.001	75.4 ± 0.16	73.72 ± 0.27	<0.001
Fasting glucose (mg/dL)	103.02 ± 0.28	106.22 ± 0.55	<0.001	106.25 ± 0.45	109.17 ± 0.9	0.003	99.72 ± 0.34	103.81 ± 0.68	<0.001
Total cholesterol (mg/dL)	197.56 ± 0.45	189.96 ± 0.75	<0.001	195.75 ± 0.66	184.1 ± 1.22	<0.001	199.4 ± 0.53	194.76 ± 0.98	<0.001
HDL-C (mg/dL)	50.39 ± 0.15	49.1 ± 0.26	<0.001	46.73 ± 0.19	46.58 ± 0.36	0.711	54.14 ± 0.2	51.16 ± 0.37	<0.001
EQ-5D index	0.96 ± 0	0.9 ± 0	<0.001	0.97 ± 0	0.92 ± 0	<0.001	0.95 ± 0	0.88 ± 0	<0.001

Values are presented as estimated % (standard error) or as the mean ± standard deviation. *p*-values were analyzed by *t*-test or χ^2^ test. Abbreviations: HGS, handgrip strength; BMI, body mass index; WC, waist circumference; SBP, systolic blood pressure; DBP, diastolic blood pressure; HDL-C, high-density lipoprotein cholesterol; EQ-5D, European Quality of Life Scale-Five index.

**Table 2:** Hand grip strength in people with or without osteoporosis.

		*N*	HGS (kg)		
			Model 1 ^a^	Model 2 ^b^	Model 3 ^c^
Total	Healthy individuals	12,465	32.31 ± 0.12	29.88 ± 0.08	29.88 ± 0.08
	Osteoporotic patients	1501	21.59 ± 0.22	30.28 ± 0.17	30.25 ± 0.18
	*p*-value		<0.001	0.028	0.049
Male	Healthy individuals	6102	39.7 ± 0.13	38.46 ± 0.11	38.46 ± 0.11
	Osteoporotic patients	97	34.18 ± 1.19	37.56 ± 0.96	37.52 ± 0.96
	*p*-value		<0.001	0.355	0.336
Female	Healthy individuals	6363	23.87 ± 0.09	23.11 ± 0.08	23.11 ± 0.08
	Osteoporotic patients	1404	20.68 ± 0.17	22.63 ± 0.16	22.62 ± 0.16
	*p*-value		<0.001	0.008	0.006

Values are presented as the mean ± standard deviation. *p*-values were analyzed using analysis of covariance. ^a^ Model 1 was unadjusted. ^b^ Model 2 was adjusted for sex, age, income, education level, smoking status, drinking status, and physical activity level. ^c^ Model 3 was adjusted for sex, age, income, education level, smoking status, drinking status, physical activity, diabetes, high blood pressure, and hypercholesterolemia.

**Table 3:** The comparison of EQ-5D index by HGS and osteoporosis.

		*N*	EQ-5D Index		
			Model 1 ^a^	Model 2 ^b^	Model 3 ^c^
Total	Healthy individuals with strong HGS	9588	0.97 ± 0.00	0.95 ± 0.00	0.95 ± 0.00
	Osteoporotic patients with strong HGS	862	0.90 ± 0.01	0.93 ± 0.01	0.93 ± 0.01
	Healthy individuals with weak HGS	2877	0.92 ± 0.00	0.93 ± 0.00	0.93 ± 0.00
	Osteoporotic patients with weak HGS	639	0.83 ± 0.01	0.87 ± 0.01	0.87 ± 0.01
	*p*-value		<0.001	<0.001	<0.001
Male	Healthy individuals with strong HGS	4602	0.97 ± 0.00	0.96 ± 0.00	0.96 ± 0.00
	Osteoporotic patients with strong HGS	50	0.94 ± 0.2	0.95 ± 0.01	0.95 ± 0.01
	Healthy individuals with weak HGS	1500	0.93 ± 0.00	0.94 ± 0.00	0.94 ± 0.00
	Osteoporotic patients with weak HGS	47	0.78 ± 0.05	0.82 ± 0.05	0.82 ± 0.05
	*p*-value		<0.001	<0.001	<0.001
Female	Healthy individuals with strong HGS	4986	0.96 ± 0.00	0.94 ± 0.00	0.94 ± 0.00
	Osteoporotic patients with strong HGS	812	0.90 ± 0.01	0.92 ± 0.01	0.92 ± 0.01
	Healthy individuals with weak HGS	1377	0.90 ± 0.00	0.91 ± 0.00	0.92 ± 0.00
	Osteoporotic patients with weak HGS	592	0.83 ± 0.01	0.87 ± 0.01	0.87 ± 0.01
	*p*-value		<0.001	<0.001	<0.001

Values are presented as the mean ± standard deviation. *p*-values were analyzed using analysis of covariance. HGS, hand grip strength; EQ-5D, European Quality of Life Scale. ^a^ Model 1 was unadjusted. ^b^ Model 2 was adjusted for sex, age, income, education level, smoking status, drinking status, and physical activity level. ^c^ Model 3 was adjusted for sex, age, income, education level, smoking status, drinking status, physical activity, diabetes, high blood pressure, and hypercholesterolemia.

## Data Availability

These data are available from a public open-access repository. The data link is https://knhanes.kdca.go.kr/knhanes/main.do, accessed on 31 October 2021.

## Supplementary Materials

The following supporting information can be downloaded at: https://www.mdpi.com/article/10.3390/medicina59122148/s1, Table S1: Bonferroni post hoc analysis for multiple comparisons of subgroups in Model 3; Table S2: Odds ratios of all five dimensions of the EQ-5D according to the presence of osteoporosis and HGS.

## References

[1] Bohannon R.W. (2019). Grip Strength: An Indispensable Biomarker For Older Adults. Clin. Interv. Aging.

[2] Yoo J.I., Choi H., Ha Y.C. (2017). Mean Hand Grip Strength and Cut-off Value for Sarcopenia in Korean Adults Using KNHANES VI. J. Korean Med. Sci..

[3] Vancini R.L., dos Santos Andrade M., Andre Barbosa de Lira C., Theodoros Nikolaidis P., Knechtle B. (2022). Is It Possible to Age Healthy Performing Ultra-endurance Exercises?. Int. J. Sport Stud. Health.

[4] Pacholek M. (2021). The Effects of Various Stimuli on Motivation and Physical Fitness of Physically Active and Non-Active Students. Ann. Appl. Sport Sci..

[5] Cheung C.L., Tan K.C., Bow C.H., Soong C.S., Loong C.H., Kung A.W. (2012). Low handgrip strength is a predictor of osteoporotic fractures: Cross-sectional and prospective evidence from the Hong Kong Osteoporosis Study. Age.

[6] Soysal P., Hurst C., Demurtas J., Firth J., Howden R., Yang L., Tully M.A., Koyanagi A., Ilie P.C., Lopez-Sanchez G.F. (2021). Handgrip strength and health outcomes: Umbrella review of systematic reviews with meta-analyses of observational studies. J. Sport Health Sci..

[7] Norman K., Stobaus N., Gonzalez M.C., Schulzke J.D., Pirlich M. (2011). Hand grip strength: Outcome predictor and marker of nutritional status. Clin. Nutr..

[8] Luna-Heredia E., Martin-Pena G., Ruiz-Galiana J. (2005). Handgrip dynamometry in healthy adults. Clin. Nutr..

[9] Ahn H., Choi H.Y., Ki M. (2022). Association between levels of physical activity and low handgrip strength: Korea National Health and Nutrition Examination Survey 2014-2019. Epidemiol. Health.

[10] Wang Y.C., Bohannon R.W., Li X., Sindhu B., Kapellusch J. (2018). Hand-Grip Strength: Normative Reference Values and Equations for Individuals 18 to 85 Years of Age Residing in the United States. J. Orthop. Sports Phys. Ther..

[11] Hajatnia B., Tajeri B., Haji-Alizadeh K. (2023). Comparing the Effectiveness of Spirituality Therapy and Acceptance and Commitment Therapy on Sleep Quality, Resilience, and Death Anxiety in the Elderly: Spirituality therapy and ACT in the elderly. Int. J. Body Mind Cult..

[12] Leboime A., Confavreux C.B., Mehsen N., Paccou J., David C., Roux C. (2010). Osteoporosis and mortality. Jt. Bone Spine.

[13] Stanghelle B., Bentzen H., Giangregorio L., Pripp A.H., Bergland A. (2019). Associations between health-related quality of life, physical function and pain in older women with osteoporosis and vertebral fracture. BMC Geriatr..

[14] Alawi M., Begum A., Harraz M., Alawi H., Bamagos S., Yaghmour A., Hafiz L. (2021). Dual-Energy X-Ray Absorptiometry (DEXA) Scan Versus Computed Tomography for Bone Density Assessment. Cureus.

[15] Luo Y., Jiang K., He M. (2020). Association between grip strength and bone mineral density in general US population of NHANES 2013–2014. Arch. Osteoporos..

[16] Siris E.S., Adler R., Bilezikian J., Bolognese M., Dawson-Hughes B., Favus M.J., Harris S.T., Jan de Beur S.M., Khosla S., Lane N.E. (2014). The clinical diagnosis of osteoporosis: A position statement from the National Bone Health Alliance Working Group. Osteoporos. Int..

[17] Musalek C., Kirchengast S. (2017). Grip Strength as an Indicator of Health-Related Quality of Life in Old Age-A Pilot Study. Int. J. Environ. Res. Public Health.

[18] Yun I., Park Y.S., Park E.C., Jang S.I. (2022). Association between changes in working status and hand-grip strength among Korean middle-aged and older adults: A longitudinal panel study. Sci. Rep..

[19] Sayer A.A., Syddall H.E., Martin H.J., Dennison E.M., Roberts H.C., Cooper C. (2006). Is grip strength associated with health-related quality of life? Findings from the Hertfordshire Cohort Study. Age Ageing.

[20] Wang M.T., Yao S.H., Wong P., Trinh A., Ebeling P.R., Tran T., Milat F., Mutalima N. (2017). Hip fractures in young adults: A retrospective cross-sectional study of characteristics, injury mechanism, risk factors, complications and follow-up. Arch. Osteoporos..

[21] Herath M., Cohen A., Ebeling P.R., Milat F. (2022). Dilemmas in the Management of Osteoporosis in Younger Adults. JBMR Plus.

[22] Tsuji S., Tsunoda N., Yata H., Katsukawa F., Onishi S., Yamazaki H. (1995). Relation Between Grip Strength and Radial Bone Mineral Density in Young Athletes. Arch. Phys. Med. Rehabil..

[23] Kweon S., Kim Y., Jang M.J., Kim Y., Kim K., Choi S., Chun C., Khang Y.H., Oh K. (2014). Data resource profile: The Korea National Health and Nutrition Examination Survey (KNHANES). Int. J. Epidemiol..

[24] Chang S.Y., Han B.D., Han K.D., Park H.J., Kang S. (2022). Relation between Handgrip Strength and Quality of Life in Patients with Arthritis in Korea: The Korea National Health and Nutrition Examination Survey, 2015–2018. Medicina.

[25] Son D.H., Yoo J.W., Cho M.R., Lee Y.J. (2018). Relationship Between Handgrip Strength and Pulmonary Function in Apparently Healthy Older Women. J. Am. Geriatr. Soc..

[26] Roberts H.C., Denison H.J., Martin H.J., Patel H.P., Syddall H., Cooper C., Sayer A.A. (2011). A review of the measurement of grip strength in clinical and epidemiological studies: Towards a standardised approach. Age Ageing.

[27] Emrani Z., Akbari Sari A., Zeraati H., Olyaeemanesh A., Daroudi R. (2020). Health-related quality of life measured using the EQ-5D-5 L: Population norms for the capital of Iran. Health Qual. Life Outcomes.

[28] Park B., Ock M., Lee H.A., Lee S., Han H., Jo M.W., Park H. (2018). Multimorbidity and health-related quality of life in Koreans aged 50 or older using KNHANES 2013-2014. Health Qual. Life Outcomes.

[29] Lee Y.-h., Choi J.-s., Rhee J.-a., Ryu S.-y., Shin M.-h., Kim J.-h. (2009). A Study on the Application of the Korean Valuation Weights for EuroQoL-5 Dimension. J. Korean Soc. Health Educ. Promot..

[30] Kim C.R., Jeon Y.J., Kim M.C., Jeong T., Koo W.R. (2018). Reference values for hand grip strength in the South Korean population. PLoS ONE.

[31] Li Y.Z., Zhuang H.F., Cai S.Q., Lin C.K., Wang P.W., Yan L.S., Lin J.K., Yu H.M. (2018). Low Grip Strength is a Strong Risk Factor of Osteoporosis in Postmenopausal Women. Orthop. Surg..

[32] Bohannon R.W. (2015). Muscle strength: Clinical and prognostic value of hand-grip dynamometry. Curr. Opin Clin. Nutr. Metab. Care.

[33] Yanai H. (2015). Nutrition for Sarcopenia. J. Clin. Med. Res..

[34] Eleni A., Andrea M., Johanna D. (2001). Nutrition and Quality of Life in Older Adults. J. Gerontol..

[35] Sakazaki T., Koike T., Yanagimoto Y., Oshida Y. (2012). Association between gait speed and bone strength in community-dwelling postmenopausal Japanese women. Environ. Health Prev. Med..

[36] Hayashida I., Tanimoto Y., Takahashi Y., Kusabiraki T., Tamaki J. (2014). Correlation between muscle strength and muscle mass, and their association with walking speed, in community-dwelling elderly Japanese individuals. PLoS ONE.

[37] Cooper R., Hardy R., Aihie Sayer A., Ben-Shlomo Y., Birnie K., Cooper C., Craig L., Deary I.J., Demakakos P., Gallacher J. (2011). Age and gender differences in physical capability levels from mid-life onwards: The harmonisation and meta-analysis of data from eight UK cohort studies. PLoS ONE.

[38] Henriksen M., Rosager S., Aaboe J., Graven-Nielsen T., Bliddal H. (2011). Experimental knee pain reduces muscle strength. J. Pain.

[39] Friden C., Thoors U., Glenmark B., Kosek E., Nordmark B., Lundberg I.E., Opava C.H. (2013). Higher pain sensitivity and lower muscle strength in postmenonpausal women with early rheumatoid arthritis compared with age-matched healthy women--a pilot study. Disabil. Rehabil..

[40] Zasadzka E., Pieczynska A., Trzmiel T., Kleka P., Pawlaczyk M. (2021). Correlation between Handgrip Strength and Depression in Older Adults-A Systematic Review and a Meta-Analysis. Int. J. Environ. Res. Public Health.

[41] Noh H.M., Park Y.S. (2020). Handgrip strength, dynapenia, and mental health in older Koreans. Sci. Rep..

[42] Kim J.H. (2019). Effect of grip strength on mental health. J. Affect Disord..

[43] Shaughnessy K.A., Hackney K.J., Clark B.C., Kraemer W.J., Terbizan D.J., Bailey R.R., McGrath R. (2020). A Narrative Review of Handgrip Strength and Cognitive Functioning: Bringing a New Characteristic to Muscle Memory. J. Alzheimer’s Dis..

[44] Fukumori N., Yamamoto Y., Takegami M., Yamazaki S., Onishi Y., Sekiguchi M., Otani K., Konno S., Kikuchi S., Fukuhara S. (2015). Association between hand-grip strength and depressive symptoms: Locomotive Syndrome and Health Outcomes in Aizu Cohort Study (LOHAS). Age Ageing.

[45] Pedersen B.K. (2013). Muscle as a secretory organ. Compr. Physiol..

