# Supplementary material for: Hand Grip Strength, Osteoporosis, and Quality of Life in Middle-Aged and Older Adults
Source: Medicina (Kaunas). 2023 Dec 11;59(12):2148. doi: 10.3390/medicina59122148 (PMC10744398; doi:10.3390/medicina59122148)
Supplement: Supplementary file 1 [file medicina-59-02148-s001.zip › medicina-2755187-supplementary.pdf]

Table S1. Bonferroni post hoc analysis for multiple comparisons of subgroups in Model 3[a]

	P-value		
	Total	Male	Female
Healthy individuals with strong HGS vs Osteoporotic patients with strong HGS	<.001*	1	<.001*
Healthy individuals with strong HGS vs Healthy individuals with weak HGS	<.001*	<.001*	<.001*
Healthy individuals with strong HGS vs Osteoporotic patients with weak HGS	<.001*	0.018	<.001*
Osteoporotic patients with strong HGS vs Healthy individuals with weak HGS	1	1	1
Osteoporotic patients with strong HGS vs Osteoporotic patients with weak HGS	<.001*	0.042	<.001*
Healthy individuals with weak HGS vs Osteoporotic patients with weak HGS	<.001*	0.066	<.001*

p-values were analyzed by Bonferroni post hoc analysis after ANCOVA.

*p<0.0083 between the two groups

HGS, hand grip strength; EQ-5D, European Quality of Life Scale.

[a]Model 3 was adjusted for sex, age, income, education level, smoking status, drinking status, physical activity, diabetes, high blood pressure, and hypercholesterolemia.

Table S2. Odds ratios of all five dimensions of the EQ-5D according to the presence of osteoporosis and HGS

		**Model 1**[a]	**Model 2**[b]	**Model 3**[c]
Total				
Mobility	Healthy individuals with strong HGS	1(reference)	1(reference)	1(reference)
	Osteoporotic patients with strong HGS	4.87(4.06–5.85)	1.85(1.50–2.29)	1.86(1.50–2.29)
	Healthy individuals with weak HGS	3.85(3.42–4.34)	1.82(1.57–2.11)	1.82(1.57–2.10)
	Osteoporotic patients with weak HGS	11.45(9.30–14.10)	2.69(2.09–3.46)	2.68(2.08–3.44)
	P-value	<.001	<.001	<.001
Self-care	Healthy individuals with strong HGS	1(reference)	1(reference)	1(reference)
	Osteoporotic patients with strong HGS	3.18(2.15–4.70)	1.38(0.92–2.07)	1.39(0.92–2.09)
	Healthy individuals with weak HGS	4.42(3.53–5.55)	2.03(1.54–2.69)	2.01(1.52–2.67)
	Osteoporotic patients with weak HGS	9.64(7.05–13.17)	2.63(1.82–3.81)	2.63(1.81–3.80)
	P-value	<.001	<.001	<.001
Usual activities	Healthy individuals with strong HGS	1(reference)	1(reference)	1(reference)
	Osteoporotic patients with strong HGS	3.75(2.91–4.83)	1.51(1.15–1.98)	1.50(1.14–1.98)
	Healthy individuals with weak HGS	3.51(2.97–4.15)	1.69(1.39–2.06)	1.68(1.38–2.05)
	Osteoporotic patients with weak HGS	9.18(7.19–11.73)	2.38(1.78–3.18)	2.36(1.77–3.15)
	P-value	<.001	<.001	<.001
Pain/discomfort	Healthy individuals with strong HGS	1(reference)	1(reference)	1(reference)
	Osteoporotic patients with strong HGS	2.76(2.36–3.24)	1.48(1.25–1.76)	1.48(1.25–1.76)
	Healthy individuals with weak HGS	1.92(1.72–2.15)	1.40(1.23–1.58)	1.39(1.23–1.58)
	Osteoporotic patients with weak HGS	4.63(3.78–5.66)	1.96(1.56–2.47)	1.96(1.56–2.47)
	P-value	<.001	<.001	<.001
Anxiety/depression	Healthy individuals with strong HGS	1(reference)	1(reference)	1(reference)
	Osteoporotic patients with strong HGS	2.70(2.15–3.38)	1.52(1.19–1.94)	1.52(1.19–1.94)
	Healthy individuals with weak HGS	1.85(1.59–2.16)	1.38(1.17–1.63)	1.38(1.17–1.63)
	Osteoporotic patients with weak HGS	3.91(3.07–4.97)	1.76(1.35–2.30)	1.75(1.34–2.29)

	P-value	<.001	<.001	<.001
Male				
Mobility	Healthy individuals with strong HGS	1(reference)	1(reference)	1(reference)
	Osteoporotic patients with strong HGS	2.25(1.03–4.88)	1.23(0.53–2.86)	1.21(0.52–2.82)
	Healthy individuals with weak HGS	4.26(3.57–5.10)	1.93(1.56–2.39)	1.92(1.55–2.38)
	Osteoporotic patients with weak HGS	14.56(6.91–30.66)	4.30(1.85–10.02)	4.25(1.85–9.76)
	P-value	<.001	<.001	<.001
Self-care	Healthy individuals with strong HGS	1(reference)	1(reference)	1(reference)
	Osteoporotic patients with strong HGS	2.84(0.66–12.19)	1.67(0.38–7.29)	1.60(0.37–6.97)
	Healthy individuals with weak HGS	3.92(2.82–5.45)	1.79(1.16–2.78)	1.79(1.15–2.77)
	Osteoporotic patients with weak HGS	19.42(7.55–49.90)	6.08(2.16–17.11)	6.03(2.23–16.35)
	P-value	<.001	<.001	<.001
Usual activities	Healthy individuals with strong HGS	1(reference)	1(reference)	1(reference)
	Osteoporotic patients with strong HGS	4.58(1.89–11.10)	2.60(1.10–6.13)	2.42(1.03–5.69)
	Healthy individuals with weak HGS	3.89(3.04–4.98)	1.81(1.34–2.46)	1.82(1.34–2.47)
	Osteoporotic patients with weak HGS	15.24(6.87–33.78)	4.33(1.88–10.00)	4.19(1.92–9.13)
	P-value	<.001	<.001	<.001
Pain/discomfort	Healthy individuals with strong HGS	1(reference)	1(reference)	1(reference)
	Osteoporotic patients with strong HGS	2.06(1.02–4.16)	1.46(0.74–2.91)	1.46(0.73–2.91)
	Healthy individuals with weak HGS	1.97(1.68–2.31)	1.31(1.08–1.60)	1.30(1.06–1.59)
	Osteoporotic patients with weak HGS	6.95(3.53–13.67)	3.10(1.59–6.02)	3.12(1.58–6.16)
	P-value	<.001	<.001	<.001
Anxiety/depression	Healthy individuals with strong HGS	1(reference)	1(reference)	1(reference)
	Osteoporotic patients with strong HGS	1.79(0.43–7.45)	1.26(0.31–5.13)	1.23(0.30–4.98)
	Healthy individuals with weak HGS	2.11(1.63–2.72)	1.43(1.05–1.94)	1.42 (1.04–1.93)
	Osteoporotic patients with weak HGS	4.17(1.58–10.99)	1.84(0.72–4.74)	1.86(0.72–4.79)
	P-value	<.001	<.001	<.001

Female				
	Healthy individuals with strong HGS	1(reference)	1(reference)	1(reference)
	Osteoporotic patients with strong HGS	4.26(3.51–5.18)	1.85(1.48–2.30)	1.85(1.49–2.30)
Mobility	Healthy individuals with weak HGS	3.50(2.97–4.13)	1.69(1.38–2.06)	1.68(1.38–2.06)
	Osteoporotic patients with weak HGS	9.49(7.56–11.91)	2.58(1.95–3.40)	2.57(1.95–3.39)
	P-value	<.001	<.001	<.001
	Healthy individuals with strong HGS	1(reference)	1(reference)	1(reference)
	Osteoporotic patients with strong HGS	3.21(2.10–4.89)	1.40(0.91–2.15)	1.43(0.96–2.20)
Self-care	Healthy individuals with weak HGS	4.98(3.66–6.79)	2.30(1.61–3.29)	2.27(1.58–3.25)
	Osteoporotic patients with weak HGS	8.95(6.25–12.80)	2.50(1.70–3.68)	2.51(1.70–3.71)
	P-value	<.001	<.001	<.001
	Healthy individuals with strong HGS	1(reference)	1(reference)	1(reference)
	Osteoporotic patients with strong HGS	3.10(2.36–4.08)	1.39(1.04–1.85)	1.40(1.05–1.86)
Usual activities	Healthy individuals with weak HGS	3.20(2.59–3.96)	1.55(1.21–2.00)	1.54(1.20–2.00)
	Osteoporotic patients with weak HGS	7.39(5.68–9.63)	2.17(1.58–2.99)	2.17(1.57–2.99)
	P-value	<.001	<.001	<.001
	Healthy individuals with strong HGS	1(reference)	1(reference)	1(reference)
	Osteoporotic patients with strong HGS	2.21(1.86–2.62)	1.54(1.29–1.85)	1.54(1.28–1.85)
Pain/discomfort	Healthy individuals with weak HGS	1.89(1.63–2.19)	1.43(1.21–1.68)	1.43(1.21–1.68)
	Osteoporotic patients with weak HGS	3.54(2.86–4.38)	2.02(1.58–2.58)	2.02(1.58–2.58)
	P-value	<.001	<.001	<.001
	Healthy individuals with strong HGS	1(reference)	1(reference)	1(reference)
	Osteoporotic patients with strong HGS	2.10(1.65–2.67)	1.56(1.21–2.02)	1.56(1.21–2.01)
Anxiety/depression	Healthy individuals with weak HGS	1.68(1.36–2.06)	1.30(1.04–1.64)	1.30(1.04–1.63)
	Osteoporotic patients with weak HGS	2.99(2.31–3.87)	1.84(1.38–2.46)	1.84(1.38–2.46)
	P-value	<.001	<.001	<.001

Values are presented as odds ratios (95% CI) and were analyzed using multiple logistic regression.

HGS, hand grip strength; EQ-5D, European Quality of Life Scale.

[a]Model 1 was unadjusted.

[b]Model 2 was adjusted for sex, age, income, education level, smoking status, drinking status, and physical activity level.

[c]Model 3 was adjusted for sex, age, income, education level, smoking status, drinking status, physical activity, diabetes, high blood pressure, and hypercholesterolemia.
